# Selenium Concentrations for Maximisation of Thioredoxin Reductase 2 Activity and Upregulation of Its Gene Transcripts in Senescent Human Fibroblasts

**DOI:** 10.3390/antiox6040083

**Published:** 2017-10-30

**Authors:** Hazem K. Ghneim

**Affiliations:** Chair of Medical and Molecular Genetics Research, Department of Clinical Laboratory Sciences, College of Applied Medical Sciences, King Saud University, Riyadh 11433, Saudi Arabia; hghneim@hotmail.com; Tel.: +966-114-693-703

**Keywords:** selenium, thioredoxin reductase 2, senescent fibroblasts, oxidative stress

## Abstract

Thioredoxin reductase 2 (TR2) activity, its gene transcripts, and hydrogen peroxide (H_2_O_2_) generation were examined in biochemically identified early-senescent P20 and senescent P30 fibroblasts subcultured in media (MEM2–MEM8) containing Se concentrations at 1.25, 2.5, 3.5, 5.0, 6.0, 7.0, and 8.0 µM, respectively. Although TR2 activity was moderately increased in P20 and P30 cells subcultured in routine growth medium (MEM1), there were progressive significant activity increases in the same cells subcultured in MEM2–MEM8. Such increases were proportional to Se concentration and peaked in P30 cells incubated with MEM7 and MEM8. H_2_O_2_ generation underwent progressive increases in MEM1-incubated P20 and P30 cells, peaking in the latter, but was gradually lowered in those incubated with MEM2–MEM8, reaching its lowest values when cells were incubated with MEM7 and MEM8. In parallel, TR2 gene transcripts underwent significant upregulation in P20 cells and higher magnitude upregulation in P30 cells subcultured in MEM2, MEM4, and MEM8 compared to those recorded for P5 pre-senescent cells subcultured in the same media. The computed Km Se values with respect to TR2 activity equaled 3.34 and 4.98 µM for P20 and P30 cells, respectively, with corresponding V_max_ activities of 55.9 and 96.2 nmol/min/mg protein. It is concluded that senescent P30 cells utilize more Se and achieve maximal TR2 activity to combat oxidative injury.

## 1. Introduction

Reactive oxygen species (ROS) consist of different forms of partially reduced oxygen and are generated intracellularly during metabolism. Most of these species are derived from the superoxide anion in the mitochondrial matrix [1,2] and are dismutated to hydrogen peroxide (H_2_O_2_) by superoxide dismutase. To this end, regulation of mitochondrial H_2_O_2_ at small concentrations is important, since the latter takes part in physiological cell signaling and prevents oxidative stress (OS) and necrosis [3]. An antioxidant enzyme system has evolved in cells to quench excessive H_2_O_2_ generation to water. Such enzymes include Glutathione peroxidase(s) (GPx), Thioredoxin reductase(s) (TR), and Catalase (CAT), which act to maintain a reducing cellular environment and to control the cellular apoptotic pathway [4].

Thioredoxin (TXN), a redox protein, is responsible for maintaining proteins in their reduced state. TXN must be in reduced form to be active and is reduced by electrons from NADPH via TR activity [5]. Human TXN and TR are expressed as isoforms that are either cytosolic (TXN1 and TR1) or mitochondrial (TXN2 and TR2), and a third form is mainly expressed in testis [6]. TR is a homodimeric selenoprotien that catalyses the transfer of electrons from NADPH via tightly bound FAD. Each monomer consists of a FAD prosthetic group, a redox active disulfide site, and a penultimate selenocysteine amino acid residue, making selenium (Se) an integral part of the active site of the enzyme [7]. Many studies have reported on the protective effects of TR against cellular stresses including growth inhibition and H_2_O_2_-induced cell death [8,9,10]. To this end, it has been demonstrated that inhibition of both TR1 and TR2 affects cellular redox balance, mitochondrial membrane permeability, and release of apoptotic factors [11].

Se is an essential metalloid incorporated as selenocysteine in many enzymes. Many selenoproteins, including TR, are involved in antioxidant defense, redox homeostasis, and signaling [12]. In addition, activation of several low molecular weight antioxidants such as Q_10_, ascorbate, and vitamin E are mediated by TR, thus making Se necessary for combating OS [13]. Many studies have reported that as Se concentration was increased in the medium of cultured cells, its incorporation into TR increased, leading to an increase in the enzyme activity and its mRNA levels. Such Se supplementation resulted in the induction of TR and GPx activities and the protection of cultured human endothelial cells against oxidative damage by lipid hydroperoxides and the enhancement of calcium signaling [14,15]. Other reports indicated that trophoblast cells derived from the placental tissue of pre-eclamptic patients incubated with sodium selenite and selenomethionine provided protection against OS and upregulation of TR and GPx expression levels [16,17]. One more study demonstrated that oxidatively stressed bone marrow stromal cells cultured in selenite supplemented medium (100 nM) restored basal TR activity and boosted the antioxidant capacity of the cells [18]. Contrary to the above, very few studies have demonstrated a connection between Se, age, and longevity both in vivo and in vitro in cultured cells. In subjects over 60, it was shown that a drop in serum Se and GPx activity occurred [19]. Other studies suggested that serum Se can be used as an indicator of longevity and that low Se levels lead to a lowered antioxidant defense [20,21]. In culture, it was shown that adrenocortical cells underwent an extension of replicative life span upon supplementation of the medium with Se [22]. In this context, we demonstrated that GPx activity was significantly increased, and coupled H_2_O_2_ generation was significantly decreased in senescent cultured human fibroblasts incubated with medium containing triple human plasma Se levels, thereby increasing the number of population doublings [23]. More recently, it was reported that Se levels regulate the entry into replicative senescence, and whereas its supplementation extends the number of population doublings, its deficiency impairs the proliferative capacity of W1-38 fibroblasts [24].

In light of the above-mentioned paucity or lack of data, this study was conducted to examine the effect of senescence on mitochondrial TR2 activity in serially subcultured human fibroblasts. In addition, the effect of increasing supplemented Se concentrations on TR2 activity and its gene expression levels in pre-senescent and senescent fibroblasts was studied. Furthermore, the optimal Km Se concentrations required to achieve maximal activation of the enzyme as well as its gene expression levels were investigated in biochemically characterized senescent fibroblasts at passages 20 and 30 of the subculture.

## 2. Materials and Methods

### 2.1. Cultivation of Primary Fibroblast Cultures

Primary human fibroblast cultures were prepared from six forearm skin biopsies (~15 mg weight) of normal young adult volunteers (average age 24.2 ± 1.51 years, range 20.2–25.1). Biopsies were excised at the Department of Anatomy, King Khaled University Hospital, Riyadh. The procedure and subject consent for obtaining the biopsies were approved by the Ethics Committee/IRB of the College of Medicine, King Saud University (approval code # 14/4001/IRB dated 5 June 2017). Confluent cultures were established by cultivating the fibroblasts in routine growth Eagles Minimum Essential Medium (MEM1) containing 10% fetal bovine serum (FBS), and cells were harvested by trypsinisation. The culture process and details related to the preparation and constituents of MEM1, as well as the trypsinisation and harvesting media, have been presented previously [25,26]. Fibroblasts were cultured in 75 cm^2^ flasks using medium (20 mL) in a Gelaire BSB4 Laminar-flow cabinet (Sydney, Australia) at 37 °C in an atmosphere containing 18% O_2_ and 5% CO_2_.

### 2.2. Preparation and Characterization of Senescent Fibroblast Cultures

Senescent fibroblast cultures were prepared by serial subculture of the six primary passage 1 cultures up to passage 30 using routine growth MEM1. Each passage number represented one population doubling as confluent cells from one 75 cm^2^ flask were trypsinised and split into two flasks. In order to identify senescent subcultures, cells at passages (P) 5, 10, 15, 20, 25, and 30 were examined for their ability to incorporate L-(U-^14^C) leucine into protein, and (methyl-^3^H) thymidne into DNA. In addition, the levels and generation rates of several oxidative stress markers associated with the onset and advancement of senescence were determined in the same subcultures. These included hydrogen peroxide (H_2_O_2_), superoxide anions (SOA), lipid peroxides (LPO), protein carbonyl content (PCC), and the reduced/oxidized glutathione ratio (GSH/GSSG). Details of methodologies regarding the assays of all the above-mentioned parameters are not presented here for lack of space, but are extensively referenced and documented by us elsewhere [23,25,27].

### 2.3. Preparation of Se-Supplemented Media

In this study, several sets of culture media were prepared. The first was routine growth medium (MEM1) containing 10% FBS as the only source of selenium, thus imparting a final concentration of the trace element equal to 0.65 µM. Seven other sets of media (MEM2–MEM8) were prepared by adding appropriate aliquots of analytical-grade sodium selenite (dissolved in sterile deionized water) to FBS-free (Se-Free) medium to give final Se concentrations equal to 1.25, 2.5, 3.5, 5.0, 6.0, 7.0, and 8.0 µM, respectively.

### 2.4. Experimental Design

To study the effect of senescence on mitochondrial TR2 activity and H_2_O_2_ generation, duplicate confluent fibroblast subcultures (*n* = 6) at P5, 10, 15, 20, 25, and 30 (cultivated in routine growth MEM1) were harvested by trypsinisation and pelleted by centrifugation at 200 *g* for 4 min. Cell pellets were then used to prepare mitochondrial sonicates for the assay of TR2 activity and H_2_O_2_ generation as described below.

To study the effect of increasing Se concentrations on mitochondrial TR2 activity and H_2_O_2_ generation in pre-senescent and senescent fibroblasts, MEM1 from duplicate flasks of confluent subcultures (*n* = 6) at P5, 20, and 30 was removed, and the cells were incubated with BSA-free (Se-supplemented) MEM2, 3, 4, 5, 6, 7, and 8 for ten hours. This incubation period was chosen in accordance with data obtained from the linearity studies of TR2 activity in the different passage subcultures incubated with MEM2 and MEM8 for increasing time periods (see result section). At the end of the ten hour incubation period, Se-supplemented media were removed and the cells were washed with phosphate buffered saline (3 mL, pH 7.4) and pelleted as described above. Pellets were then used to prepare mitochondrial sonicates for the assay of TR2 activity and H_2_O_2_ generation.

### 2.5. Linearity of TR2 Activity with Respect to Time in Subcultures at Passages 5, 20, and 30 Incubated with MEM2 and MEM8

To achieve maximal TR2 activity with respect to incubation time with Se-supplemented media, spent routine MEM1 of duplicate confluent subcultures at P5, 20, and 30 was replaced with FBS-free MEM2 and MEM8 containing 1.25 µM and 8 µM Se, respectively, and cells incubated for 1, 2, 4, 6, 8, 10, and 12 h. Harvested pellets were then used to prepare mitochondrial sonicates for the assay of TR2 activity. P5, 20, and 30 cells were chosen since data (presented later) indicated that those at passage 5 were identified as pre-senescent and primary, whereas those at P20 and 30 were identified as early-senescent and late-senescent, respectively. The use of MEM8 containing the highest Se concentration is justified because it gave highest TR2 activity in cells at P20, 25, and 30 (see result section).

### 2.6. Separation of Fibroblast Mitochondria

Harvested pellets from ten flasks of P5, 20, and 30 cells subcultured to confluence in MEM1 and further incubated with Se-supplemented MEM2–MEM8 for 10 h, were homogenized in Mannitol solution (0.3 M, 8 mL) containing 1 mM EDTA and 10 mM Hepes buffer (pH 7.2). The homogenates were then centrifuged at 2000 rpm to 7 × 10^6^ g/min, and supernatants were collected. Debris were then resuspended in Mannitol (4 mL) and centrifuged as mentioned above, and both supernatants (8 + 4 mL) were combined and centrifuged at 10,000 rpm to 2.95 × 10^8^ g/min, and mitochondrial pellets were collected. Finally, pellets were sonicated for 20 s in ice by a Thermo-Fisher sonic dismembranator model 150 (Waltham, MA, USA) in 0.1 M Phosphate buffer (pH 7.2), and microliter aliquots were used to assay TR2 activity and H_2_O_2_ generation rates.

### 2.7. TR2 Assay

TR2 activity was measured using the enzyme’s colorimetric assay kit (Item# 10007892, Cayman Chemicals, Ann Arbor, MI, USA). The procedure is based on the catalytic reduction of 5,5’-dithio-bis (2-nitrobenzoic acid) (DTNB) by TR, which is coupled to the oxidation of one molecule of NADPH and associated with the conversion of DTNB into 5, thio-2-nitrobenzoic acid (TNB). The reaction mixture consisted of fibroblast mitochondrial sonicate (50 µL), 0.2 mM NADPH, 10 mM EDTA, and 0.2 mg/mL fetal bovine serum, and was adjusted to a total volume of 500 µL with 50 mM potassium phosphate buffer pH 7.4. The reaction was initiated by the addition of 25 mM DTNB and the change in absorbance caused by TNB formation was monitored at 405 nm. The assay was performed in the presence and absence of aurothiomalate (20 µM), an inhibitor of TR2 activity, thus allowing for correction of the activity of none-thioredoxin NADPH oxidoreductases also capable of reducing DTNB. Finally, TR2 enzymatic activity was expressed as nmole of TNB formed/min/mg protein content of the sonicate.

### 2.8. H_2_O_2_ Generation Rates

H_2_O_2_ generation rates were assayed using the method described by Zhou et al. [28] and modified by us [29]. The reaction mixture contained peroxidase dissolved in Kreb’s Ringer buffer (10 IU/mL, 100 µL), sodium phosphate buffer (50 mM, pH 7.4), and appropriately diluted standards and mitochondrial sonicates (50 µL). After incubation of the mixture for 30 min at room temperature, the reaction was initiated by the addition of ARR (Amplex Red Reagent, 10-acetyl-3,7-dihydrophenoxazine, 10 mM, 50 µL), which reacts with H_2_O_2_ to form the red fluorescent product resorufin. Fluoreseence was then monitored at E_530_ and E_590_.

### 2.9. Gene Expression Profiling of hsTR2 Using Real-Time Quantitative PCR (RT-qPCR)

Fresh pellets of primary P5 fibroblasts and senescent fibroblasts at P20 and 30 incubated in MEM2, MEM4, and MEM8 were stored in RNAlater^®^ Stabilization Solution (Qiagen, Hilden, Germany) at −80 °C. Pellets were later homogenized in a Tissue Lyser LT (Qiagen, Hilden, Germany) in 1 mL TRIzol^®^ Reagent (Invitrogen, Paisley, UK), and total RNA was extracted by standard methods. Genomic DNA was then eliminated and cDNA synthesized from RNA (1 µg) in a final reaction volume (20 µL) using the QuantiTect Reverse Transcriptase Kit (Qiagen, QuantiTect^®^, (Hilden, Germany) according to the manufacturer’s instructions. The resultant diluted cDNA (1:10, 5 µL) was then used to perform RT-qPCR by the QuantiTect SYBR-Green PCR kit (Qiagen, Hilden, Germany) and the TR2 Qiagen Primer Assay (Hs_TXNRD2_1_SG QuantiTect Primer Assay (QT00070371) in a final reaction volume (25 µL) containing the cDNA sample (5 µL), 2× SYBR Green PCR Master mix (12.5 µL), each forward and reverse primer (10 µM stock, 2.5 µL), and RNAase-free water (2.5 µL). The amplification program and PCR amplicon specificity were performed and assessed as documented by us previously [30]. Each fibroblast tissue sample was represented by biological replicas and three technical replicas, with the inclusion of a no-template control. Raw data were analyzed using the Rotor-Gene cycler Q software version 2.3 (Qiagen, Hilden, Germany) to calculate the threshold cycle using the second derivative maximum. The relative gene expression level (fold change) was determined after normalization to the expression levels of *18S* as a housekeeping gene, as previously described [31]. Fold change values were then subjected to student’s *t*-test to identify significant differences between senescent cultures against pre-senescent P5 primary cultures (*p* < 0.05 was considered as statistically significant).

### 2.10. Other Assays and Statistical Analysis

Se concentration in routine growth MEM1 was assayed by Atomic Absorption Spectrophotometry (Perkin-Elmer 2380, Waltham, MA, USA) using the appropriate wavelengths, standards, and control samples, as previously documented [32]. Total protein content of mitochondrial fibroblast sonicates (20 µL) was assayed according to Bradford [33]. One way analysis of variance (ANOVA) followed by Bonferroni correction was performed to evaluate statistical differences between mean ± SD values of TR2 activities and H_2_O_2_ generation rates using the SPSS version 17.0 (IBM, Armonk, NY, USA). Multiple comparisons were performed between sets of data of different passages cultured in different media, and *p* values < 0.05 were considered statistically significant.

## 3. Results

### 3.1. Se Concentration in Culture Media

The Se concentration in pooled batches of routine MEM1 containing 10% FBS as the only Se source equaled 0.65 ± 0.04 µM (mean ± SD of triplicate determinations). This concentration is equivalent to half the metalloid levels in normal serum. The sodium selenite-supplemented FBS-free media prepared for this study (MEM2, 3, 4, 5, 6, 7, and 8) contained 1.25, 2.5, 3.5, 5.0, 6.0, 7.0, and 8.0 µM Se, respectively. Hence MEM2 contained the lowest concentration of supplemented Se, which was approximately equivalent to the metalloid’s normal human serum levels, and MEM8 contained the highest concentration, which was equivalent to about 6 times its normal human serum levels.

### 3.2. Identification of Pre-Senescent and Senescent Fibroblast Subcultures.

#### 3.2.1. Growth and Replication of Subcultures.

Results related to the growth and replication of subcultures (used as markers of senescence) are not presented here for lack of space. Briefly, data indicated that MEM1-subcultured fibroblasts at P5 and 10 required 24 h post-subculture to achieve maximal rates of L-(U-^14^C) leucine incorporation into protein and (methyl-^3^H) thymidine incorporation into DNA and reach confluence. However, cells at P15 required 48 h, those at P20 and 25 required 96 h, and those at P30 required 120 h to reach such rates. Results also showed that once reached, maximal rates were maintained at very similar values at all longer culture periods for cells at all passages. Furthermore, very similar results were obtained for cells at all passages subcultured in all Se-supplemented media (MEM2–MEM8). The above-mentioned results are in very close agreement with data extensively documented by us for human skin fibroblast subcultures used in previous investigations [23,25,34]. Hence, throughout the current study, cells at different passages were harvested at the appropriately indicated time periods post-confluence and were replenished with fresh medium when subcultured for longer than 48 h.

#### 3.2.2. Oxidative Stress Markers are Indicative of Senescence

As evident from Table 1 data, there were no significant changes in the generation rates and levels of the oxidants H_2_O_2_, SOA, LPO, and PCC in mitochondria of P5 and P10 cells subcultured in MEM1. However, there were moderate but statistically significant increases in the values of all the examined oxidants in P15 cells compared to those documented for P5 cells (*p* < 0.05 for all comparisons). Such increases became highly significant in P20 cells (*p* < 0.001) and extremely significant and peaked in value in cells at P25 and P30 (*p* < 0.0001) when compared to those for P5 cells. Concurrently, although the GSH/GSSG ratio did not change in P5 and P10 cells, it underwent moderate but significant reductions in P15 cells (*p* < 0.05) and highly significant reductions in P20 cells (*p* < 0.001), which became extremely significant (*p* < 0.0001) in P25 and P30 cells when all ratios were compared against those for P5 cells. The above results indicated that cells at P20, P25, and P30 became oxidatively stressed; a phenomenon closely associated with proliferative senescence.

In light of the results regarding growth, replication, and oxidative stress markers, P5 subcultures were considered in this study to represent young pre-senescent fibroblasts and were considered as controls. Additionally, P20 cells were considered to be at an early phase of senescence and those at P25 and P30 as senescent.

#### 3.2.3. Effect of Senescence on Mitochondrial TR2 Activity in MEM1-Incubated Cultures

**Figure 1:**
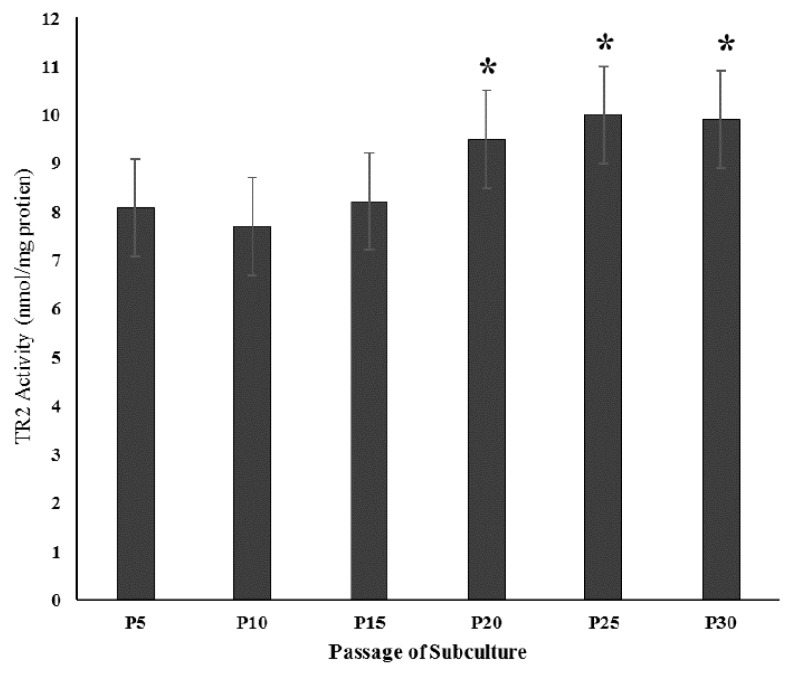
Effect of Senescence on mitochondrial TR2 activity fibroblasts subcultured in MEM1. TR2 = Mitochondrial Thioredoxin Reductase 2. Values shown are means ± SEM of duplicate determinations for 6 cultures. MEM1 = routine growth medium. * = *p* < 0.05 upon comparison of TR2 activities in P20, P25, P30 fibroblasts against those documented for P5 fibroblasts.

As seen from Figure 1, although there were no significant changes in TR2 activity in pre-senescent cells at P5, P10, and P15, there were slight but significant increases of similar magnitude in the enzyme activity in senescent P20, P25, and P30 cells. Activities equaled 8.08 ± 0.44, 7.71 ± 0.45, 8.22 ± 0.55, 9.50 ± 0.58, 10.0 ± 0.84, and 9.90 ± 0.82 nmol/min/mg protein for P5, 10, 15, 20, 25, and 30 cells, respectively (*p* < 0.05 upon comparison of the activities in senescent P20, 25, and 30 cells against that of pre-senescent P5 young controls).

#### 3.2.4. Effect of Increased Incubation Time of Pre-Senescent (P5) and Senescent (P20 and 30) Cultures with MEM2 and MEM8 on TR2 Activity

Table 2 data indicated that pre-senescent P5 fibroblasts incubated with either MEM2 (containing 1.25 µM Se equivalent to normal human plasma levels of the cation) or MEM8 (containing the highest supplemental Se concentration; 8 µM), required 4 h to achieve maximal TR2 activity. However, early-senescent P20 cells required 6 h to do so, and senescent P30 cells required 8 h. In addition, very similar maximal TR2 activities were maintained for all passage cells investigated, whether incubated with MEM2 or MEM8 for up to 12 h. Hence, to ensure maximal enzyme activity, cells at all passages in all subsequent experiments were incubated with all Se-supplemented media (MEM2–MEM8) for 10 h prior to harvesting. Closer examination of Table 2 data also showed that whereas early senescent P20 cells had a maximal TR2 activity of 37.9 ± 2.35 nmol/min/mg protein after 6 h incubation with MEM8, senescent P30 cells showed a significantly higher enzyme activity 8 h-post incubation with the same medium equal to 54.7 ± 3.34 nmol/min/mg protein. These values were significantly higher (*p* < 0.0001) when the same cells were incubated with MEM2 for the same time periods (15.6 ± 0.98 and 19.4 ± 1.18 nmol/min/mg protein for P20 and P30 cells, respectively). Such data suggested that increasing the Se concentration of the culture medium lead to stimulation of TR2 activity that progressed in value as the cells passed from early senescence to senescence. Detailed data regarding this phenomenon is presented below.

### 3.3. Effect of Increasing Se Concentrations on TR2 Activity and H_2_O_2_ Generation Rates in Pre-Senescent (P5) and Senescent (P20 and 30) Fibroblast Cultures

#### 3.3.1. TR2 Activity

As evident from Table 3, there were no significant changes in TR2 activity of pre-senescent P5 cells subcultured in MEM1or any of the Se-supplemented media. However, in early-senescent P20 cells, the enzyme activity underwent progressive and extremely significant increases in subcultures incubated with MEM2, 3, 4, 5, 6, 7, and 8 when all values were compared against those documented in the MEM1-subcultured cells (*p* < 0.0001 for all comparisons). In addition, the activity seemed to peak in P20 cells subcultured in MEM6 and was maintained at very similar levels in the cells subcultured in MEM7 and 8. As is also evident from Table 3 data, although there was an extremely significant increase in TR2 activity in MEM3-incubated P20 cells compared to those recorded for MEM2 (*p* < 0.0001), the increases were lower in magnitude but nonetheless significant as the Se concentration of the medium increased (See Table 3 *p* values). Furthermore, comparison of the enzyme activities in P20 cells incubated with MEM6 against MEM5 was not significant (*p* = 0.120). These results indicated a saturation kinetics phenomenon of TR2 activity with respect to Se concentration. Other Table 3 results showed that for senescent P30 cells there were extremely significant statistical increases in TR2 activity when fibroblasts were incubated in all Se-supplemented media compared to those recorded for the cells incubated in MEM1 (*p* < 0.0001 for all comparisons). Similar to P20 cells, the enzyme activity increases in P30 cells were progressive in nature, relative to Se in the medium, peaked in value when the cells were incubated in MEM6, and were maintained at very similar values to those incubated in MEM7 and MEM8 (see Table 3 *p* values). However, further analysis of Table 3 data revealed that much higher TR2 activities were achieved in senescent P30 cells incubated with MEM6, 7, and 8 (53.7 ± 4.78, 55.6 ± 5.22, and 55.3 ± 5.09 nmol/min/mg protein, respectively) than those achieved in early-senescent P20 cells incubated with the same media (36.4 ± 3.20, 38.5 ± 3.71, and 38.4 ± 3.69 nmol/min/mg protein, respectively; *p* < 0.0001 for all comparisons). In terms of percentage increases compared to the enzyme activities recorded for MEM1-incubated P20 and P30 cells, these amounted to ~400% and ~550%, respectively.

#### 3.3.2. H_2_O_2_ Generation

As presented in Table 4, there were no significant changes in H_2_O_2_ generation rates in pre-senescent P5 cells subcultured in any of the Se-supplemented media compared to those subcultured in MEM1. Results also indicated that H_2_O_2_ generation in MEM2-incubated early-senescent P20 cells did not significantly change upon comparison with that obtained for MEM1-incubated cells. However, rates underwent very significant reductions in the MEM3-incubated cells and extremely significant ones upon incubation in each of MEM4, 5, 6, 7, and 8 compared to those generated in MEM1-incubated cells (*p* < 0.0001 for all comparisons). Furthermore, the H_2_O_2_ generation rate reductions were progressive and peaked in magnitude when the P20 cells were incubated in MEM7 and MEM8 (4.64 ± 0.38 and 4.22 ± 0.34 pmol/min/mg protein, respectively) compared to 7.80 ± 0.63 pmol/min/mg protein generated in MEM1-incubated cells; an approximate reduction of 40% and 45%, respectively. In contrast, H_2_O_2_ generation rates in senescent P30 cells underwent very significant reductions upon incubation of the subcultures in all Se-supplemented media (MEM2–MEM8) compared to MEM1 (*p* < 0.0001 for all comparisons). Such rate reductions were progressive, relatively high in magnitude, and significant when comparisons were made between P30 cells incubated in MEM3 against MEM2, MEM4 against MEM3, MEM5 against MEM4, MEM6 against MEM5, MEM7 against MEM6, and MEM8 against MEM7 (see Table 4 *p* values). Such reductions approximately amounted to 25%, 34%, 41%, 49%, 58%, 66%, and 69% for the cells incubated in MEM2, 3, 4, 5, 6, 7, and 8, respectively, compared to those incubated in MEM1. Thus, it seems that the H_2_O_2_ generation rate reductions in senescent P30 cells peaked in value when the cells were incubated in MEM7 and 8, and were significantly higher than those noted for early-senescent P20 cells incubated with the same media, which only equaled about 40% and 45%, respectively, of the rate recorded when the cells where incubated with MEM1.

### 3.4. Effect of Increasing Se Concentrations on TR2 Gene Expression Levels in Pre-Senescent (P5), Early-Senescent (P20) and Senescent (P30) Subcultures

**Figure 2:**
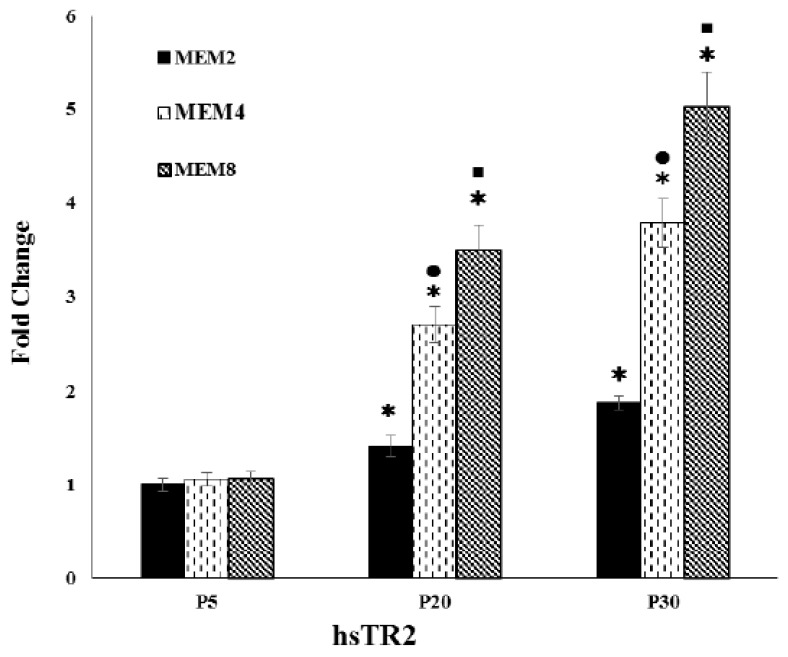
Relative gene expression of *hsTR2* in pre-senescent (P5) and senescent (P20 and P30) fibroblasts subcultured in MEM2, MEM4 and MEM8. *hsTR2* = *homo sapiens* Mitochondrial Thioredoxin Reductase. Fold change values shown are means ± SD for duplicates of the 6 cultures. Se concentration of MEM2, MEM4, and MEM8 are as documented in the text. * = *p* < 0.0001 upon comparison of the fold change in the gene expression levels of *hsTR2* in P20 and P30 cells subcultured in MEM2, MEM4, and MEM8 relative to those recorded for P5 cells cultured in the same media. ^●,■^ = *p* < 0.0001 upon comparison of the fold change values in *hsTR2* gene expression levels of P20 and P30 cells subcultured in MEM4 against MEM2 (^●^) and those subcultured in MEM8 against MEM4 (^■^).

As evident from Figure 2, there were no significant changes in *hsTR2* gene expression levels in P5 fibroblasts regardless of whether the cells were subcultured in MEM2, MEM4, or MEM8. However, in early senescent P20 cells, TR2 transcripts were very significantly upregulated by 41.3 ± 3.09%, 152.1 ± 12.2%, and 252.8 ± 17.7 (fold change values of 1.41 ± 0.11, 2.70 ± 0.19, and 3.5 ± 0.26), when the cells were subcultured in MEM2, MEM4, and MEM8, respectively, compared to transcripts recorded for P5 cells subcultured in the same media (*p* < 0.0001 for all comparisons). In P30 senescent fibroblasts subcultured in MEM2, MEM4, and MEM8, results indicated that transcripts underwent upregulation to even higher levels equaling 87.3 ± 6.98%, 246.3 ± 18.8%, and 369.3 ± 26.9% (fold change values of 1.87 ± 0.13, 3.78 ± 0.28 and 5.02 ± 0.36), respectively, compared to those recorded for P5 cells subcultured in the same media (*p* < 0.0001 for all comparisons). Further analysis of data showed that the noted upregulation of TR2 transcripts in P20 and P30 cells were very significant in cells subcultured in MEM4 against MEM2 and in MEM8 against MEM4 (*p* < 0.0001 for all comparisons).

### 3.5. Determination of Se Km Values with Respect to TR2 Activity in Senescent Fibroblasts

**Figure 3:**
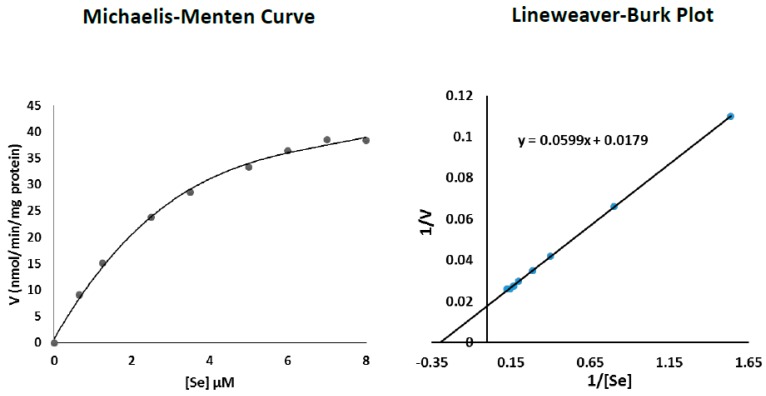
Determination of Se Km values with respect to TR2 activity in early senescent P20 cells.

**Figure 4:**
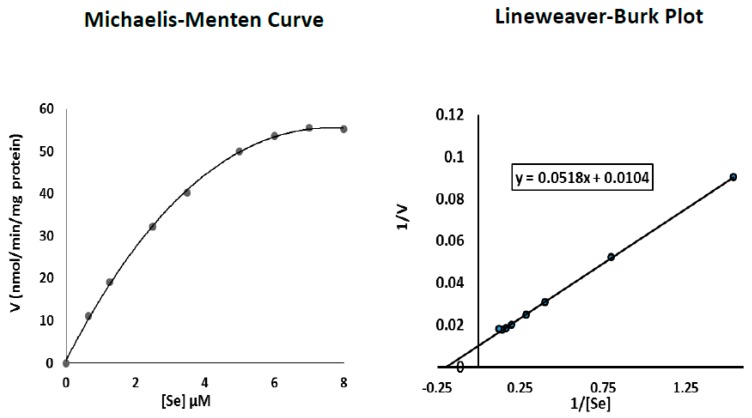
Determination of Se Km values with respect to TR2 activity in senescent P30 cells.

Data related to the effect of increasing Se concentrations on TR2 activities in early senescent P20 and senescent P30 cells (Table 3) was used to construct Michaelis-Menten and Lineweaver-Burk plots using Microsoft^®^ Excel software (Figure 3 and Figure 4). As evident from the Michaelis-Menten curves, the effect exhibited a saturation kinetics phenomenon for both cell passages. In addition, the computed data of the Lineweaver-Burk plots indicated that Se Km values equaled 3.34 and 4.98 µM for cells at P20 and P30, respectively, with corresponding Vmax TR2 activities equal to 55.9 and 96.2 nmol/min/mg protein.

## 4. Discussion

The replicative senescence of serially subcultured human fibroblasts provides a sound experimental tool for the study of the changes of various cellular aspects related to organismal aging [35,36]. Such changes include metabolic ones associated with deficiency of the antioxidant capacity in senescent cultured human fibroblasts previously studied in our laboratory [25,37]. In this study, TR2 activity and its gene expression levels were investigated in biochemically characterised pre-senescent and senescent fibroblast subcultures subjected to increasing Se concentration. The only source of Se in routine growth medium (MEM1) comes from fetal bovine serum, which provided the cells with approximately half the Se concentration of normal human plasma. The subcultures were incubated with serum-free media (MEM2–MEM8) containing Se up to about 6x the normal human plasma levels of the cation. This ensured the structural integrity of TR2 and achieved maximal antioxidant catalytic activity of the enzyme. Furthermore, this allowed enhancement of the proliferative capacity of the subcultures and increased the number of the population doublings. To this end, it has been reported that routine culture medium does not have sufficient amounts of many trace elements [38]. In addition, a recent study showed that Se supplementation of the medium selectively modulated selenoprotein expression and extended the number of population doublings of WI-38 human fibroblasts, whereas its deficiency impaired the proliferative capacity of the cells [24]. Other optimal culture conditions were provided in this study to allow fibroblasts to multiply and metabolise at maximal rates. These included provision of sufficient culture medium, addition of Hepes buffer to the culture and trypsinisation media, and the use of antibiotics to combat bacterial infection. This achieved a low percentage of cell death (~5%), unaffected by the passage number of the subcultures or the highest Se concentration used (8 µM). Preliminary data, however, showed that cell toxicity and death occurred at a Se concentration of 11 µM.

Replicative senescence at increased passages of subcultured cells has been associated by us and others with a variety of morphological, histological, and biochemical changes. This includes increased cell size, flattened cells, progressive lowered rates of cellular growth and replication, an increased rate of protein oxidant, telomere shortening, increased oxidant generation, and an increase in the number of cells exhibiting senescence associated ß- galactosidase activity [23,25,34,35,36,37,38]. In the present study, senescent subcultures were identified by examining their growth and replication rates, as well as the levels and generation rates of potent oxidants including H_2_O_2_, SOA, LPO, PCC, and the GSH/GSSG ratio. Results revealed that MEM1-cultured fibroblasts at P5 and P10 needed 24 h to become confluent and achieve maximal rates of radiolabelled thymidine and leucine incorporation into DNA and protein. In contrast, P15, P20, and P30 cells required 48, 96, and 120 h, respectively, and were thus harvested at such times post-culture (data not shown for lack of space). These harvesting times were identical with those extensively published by us previously [23,25,34], and indicated that whereas P5 and P10 cells were pre-senescent, P20 were at an early phase of senescence, and those at P30 were senescent. Harvesting subcultures at these appropriate times ensured that their DNA and protein yields were not influenced by senescence, and that any variations in TR2 activity could be contributed to senescence. Protein and DNA contents of mitochondrial sonicates of the six cultures equaled 61.3 ± 6.63 and 4.09 ± 0.46 µg/100 µL, respectively, with a protein/DNA ratio of 15.1 ± 0.45 for all passages. Other data used to identify senescent fibroblasts (Table 1) indicated very significant progressive increases in the generation rates and levels of H_2_O_2_, SOA, LPO, and PCC as the cells were subcultured beyond P15, reaching a 4-fold level increase in P30 cells compared to P5 cells. In contrast, however, there were very significant decreases in the GSH/GSSG ratio in cells beyond P15, reaching maximal low values in P25 and P30. All the above results further suggested that, whereas cells at P5 and P10 were pre-senescent, those at P20 and P30 were early-senescent and senescent, respectively, and were treated as such in all subsequent experiments.

There is a distinct lack of literature data related to the effect of senescence on TR2 activity in cells cultured in routine growth medium. Results of the present study (Figure 1) indicated very similar TR2 activities in pre-senescent P5, P10 and P15 fibroblasts subcultured in MEM1. This suggested that a near normal human plasma Se concentration (0.65 µM) was sufficient for the expression of normal TR2 activity in pre-senescent cells. However, Figure 1 data showed that there were moderate but statistically significant increases in the enzyme activities in early-senescent P20 and senescent P30 cells subcultured in MEM1 compared to those obtained for P5 pre-senescent cells (*p* < 0.05). This seemed to be a natural consequence of senescence where TR2 activity increased in an attempt to quench excessive H_2_O_2_ generation. However, such moderate increases were not sufficient to neutralise the highly excessive H_2_O_2_ generation documented for P20 and P30 subcultures in Table 1, and could have been related to the limited Se concentration of MEM1. In support of this, Table 3 results showed highly significant progressive TR2 activity increases in early-senescent P20 and senescent P30 cells incubated with MEM2–MEM8 containing increasing supplemental Se concentrations, which could not be seen when the cells were subcultured MEM1. Such increases were relative to the Se concentration, and peaked when the senescent cells were incubated with MEM7 and MEM8 containing 7.0 and 8.0 µM Se. This indicated that P20 and P30 fibroblasts were needed to combat oxidative injury by utilising higher Se concentrations for the stimulation of TR2 activities. The above results are in agreement with previous reports where Se supplementation resulted in the induction of TR2 activity and protection against oxidative stress in cultured human endothelial cells [14,15] and bone marrow stromal cultured cells [18]. More data of the present study revealed that early-senescent P20 and senescent P30 fibroblasts cultured in MEM1 generated approximately 2.5 and 4 times more H_2_O_2_ than pre-senescent P5 cells subcultured in the same medium (Table 4). However, the generated H_2_O_2_ rates were progressively and significantly reduced as the Se concentration of the medium was increased and reached lowest values in P20 and P30 cells incubated with MEM7 and MEM8. Although such lowered rates were still significantly higher (by about 30%) than those generated by P5 cells incubated with MEM7 and MEM8, they further suggest that senescent cells become resistant to oxidative stress in the presence of 7.0 and 8.0 µM Se. In this context, we have been able to demonstrate highest Glutathione peroxidase activity and concurrent lowest H_2_O_2_ generation rates in senescent fibroblasts subcultured in medium containing triple human plasma Se concentrations [23].

Parallel with the above-mentioned TR2 activity increases in P20 and P30 cells, there were very significant increases in the enzyme’s transcripts which were upregulated when the cells were incubated with Se-supplemented media (Figure 2). This is in agreement with the findings of several studies that reported upregulation of TR2 and glutathione peroxidase gene transcripts in oxidatively stressed human endothelial cells [14,15], trophoblast cells derived from placental tissue of pre-eclamptic patients [16,17], and bone-narrow stromal cells [18], all cultured in the presence of supplemental Se. What is unique to the current study, however, is that the fold change increases were proportional to the Se concentration and higher in magnitude in P30 cells compared to P20 cells. Such fold change increases equaled 41.3 ± 3.09%, 158.1 ± 12.5%, and 196.7 ± 14.9% for early-senescent P20 cells, and 85.2 ± 6.63%, 268.1 ± 21.3%, and 393.6 ± 30.1% for senescent P30 cells subcultured in MEM2, MEM4, and MEM8, respectively, compared to pre-senescent P5 cells subcultured in the same media (Figure 2). In addition, such TR2 transcript upregulation values correlated well with the corresponding increases in the biochemical activities of the enzyme noted for the same cells incubated with the same media (Table 3). The present findings further suggest that senescent P20 and P30 cells underwent endogenous oxidative stress but acquired the ability to resist this through upregulation of TR2 transcripts and the maximization of the enzyme’s catalytic activity in the presence of 7 and 8 µM Se concentrations.

Knowledge regarding the mechanisms of cellular Se uptake is very limited but likely occurs via multiple membrane transporters, which vary according to its chemical form. In human erythrocytes, anion exchanger 1 (AE1) was shown to be involved in selenite transport [39]; it also facilitates the transport of the phosphate, sulfate, and bicarbonate. In another study, the monocarboxylate transporter Jen1P was shown to transport selenite in yeast [40]. Furthermore, it was demonstrated that selenite has a lower transport rate than its reduced form in yeast [41]. Although Se uptake by fibroblasts (for which no data is available) is not a focal issue of this study, Table 2 data indicated that whereas MEM2 and MEM8 incubated pre-senescent P5 cells required 4h to maximise TR2 activity, early-senescent P20 and senescent P30 cells required 6 and 8 h, respectively. In addition, such maximal enzymes activities were maintained when cells were incubated with the same media for up to 12 h. This suggested that Se uptake by cultured fibroblasts is a time-dependent saturable process which could be transporter-mediated. Furthermore, Table 2 results indicated that P20 and P30 cells incubated with MEM8 (8.0 µM Se) for 6 and 8 h, respectively, exhibited enhanced and much higher TR2 activities compared to those documented when the cells were incubated with MEM2 (1.25 µM Se) for the same time periods. This suggested that Se uptake by cultured fibroblasts is also dose-dependent, and further supports the notion that Se uptake and utilisation is significantly increased in early-senescent P20 cells and maximised in P30 senescent cells near the end of their proliferative life span. To this end, results of the present study showed that the Km values of Se with respect to TR2 activity equaled 3.34 and 4.98 µM for P20 and P30 cells, respectively, with corresponding TR2 Vmax values of 55.8 and 96.1 nmol/min/mg protein (Figure 3 and Figure 4).

## Figures and Tables

**Table 1:** Levels and generation rates of oxidative stress markers in routine growth medium (MEM1)-serially subcultured fibroblasts.

Fibroblast Cultures (*n* = 6)/Passage of Subcultures	Oxidative Stress Markers				
	H_2_O_2_	SOA	LPO	PCC	GSH/GSSG
	pmol/min/mg protein	µmol/min/mg protein	pmol/min/mg protein	nmol/mg	
P5	3.41 ± 0.29	1.03 ± 0.06	71.6 ± 5.63	4.88 ± 0.40	61.3 ± 3.98
P10	3.28 ± 0.26	0.97 ± 0.06	73.5 ± 5.68	4.91 ± 0.41	59.6 ± 3.75
P15	3.85 ± 0.33 *	1.12 ± 0.07 *	81.7 ± 6.29 *	5.56 ± 0.46 *	55.1 ± 3.65 *
P20	7.80 ± 0.63 **	1.27 ± 0.09 **	87.4 ± 6.69 **	6.01 ± 0.47 **	51.2 ± 3.24 **
P25	12.6 ± 1.10 ***	3.95 ± 0.24 ***	272.3 ± 20.1 ***	10.8 ± 0.85 ***	28.3 ± 1.70 ***
P30	13.8 ± 1.14 ***	4.14 ± 0.25 ***	281.7 ± 21.4 ***	11.3 ± 0.86 ***	29.5 ± 1.71 ***

H_2_O_2_ = Hydrogen peroxide, SOA = Superoxide anions, LPO = Lipid peroxides, PCC = Protein carbonyl content, GSH/GSSG = Reduced/Oxidised glutathione ratio. Values shown are means ± SD of duplicate determinations for 6 cultures. MEM1 = routine growth medium. * = *p* < 0.05, ** = *p* < 0.001, *** = *p* < 0.0001 upon comparison of all values in P15, P20, P25, and P30 cells against those of P5 cells.

**Table 2:** Effect of increased incubation time of pre-senescent (P5) and senescent (P20 and P30) cultures with MEM2 and MEM8 on Thioredoxin reductase 2 (TR2) activity.

Incubation Time (Hours)	Subculture Passages TR2 Activity (nmol/min/mg protein)					
	P5		P20		P30	
	MEM2	MEM8	MEM2	MEM8	MEM2	MEM8
1	6.01 ± 0.39	6.10 ± 0.41	9.22 ± 0.55	22.4 ± 1.30	9.60 ± 0.57	23.3 ± 1.37
2	6.94 ± 0.428 *	6.91 ± 0.43 *	10.6 ± 0.67 *	28.2 ± 1.72 **	12.1 ± 0.74 **	27.1 ± 1.66 *
4	8.09 ± 0.48 **	8.12 ± 0.53 **	12.4 ± 0.75 **	32.1 ± 1.93 **	14.3 ± 0.87 **	36.8 ± 2.28 **
6	8.03 ± 0.49	8.10 ± 0.49	15.6 ± 0.98 **	37.9 ± 2.35 **^,^^■^	16.6 ± 0.98 **	45.6 ± 2.87 **
8	7.98 ± 0.49	7.94 ± 0.48	15.4 ± 0.93	38.1 ± 2.29	19.4 ± 1.18 **	54.7 ± 3.34 **^,^^■^
10	8.12 ± 0.52	7.99 ± 0.49	15.1 ± 0.91	38.4 ± 2.36	19.2 ± 1.13	55.3 ± 3.31
12	8.02 ± 0.49	8.08 ± 0.52	15.0 ± 0.92	38.3 ± 2.31	19.5 ± 1.19	56.0 ± 3.34

TR2 = Mitochondrial Thioredoxin Reductase. Values are means ± SD of duplicate determinations for 6 cultures. MEM2 and MEM8 = fetal bovine serum (FBS)—free culture media containing 1.25 and 8.0 µM Se, respectively. * = *p* < 0.01,** = *p* < 0.0001 when comparing TR2 activities in cultures incubated with MEM2 and MEM8 for 2 and 4 h (at P5), 2, 4, and 6 h (at P20), and 2, 4, 6, and 8 h (at P30) against their respective activities recorded at 1 h. ^■^ = *p* < 0.0001 when comparing TR2 activities in P20 and P30 cells incubated for 6 and 8 h, respectively, with MEM8 against those recorded for the cells incubated with MEM2 for the same periods.

**Table 3:** Effect of Se supplementation on mitochondrial TR2 activity in pre-senescent (P5) and senescent (P20 and P30) fibroblasts subcultures.

Passage of Subculture (*n* = 6)	TR2 Activity (nmol/min/mg protein)							
	MEM1	MEM2	MEM3	MEM4	MEM5	MEM6	MEM7	MEM8
P5	8.08 ± 0.78	8.31 ± 0.80	8.19 ± 0.79	7.92 ± 0.75	7.81 ± 0.74	8.11 ± 0.79	8.36 ± 0.81	8.22 ± 0.80
P20	9.50 ± 0.90	15.1 ± 1.38 *	23.8 ± 2.25 *^,■^	28.6 ± 2.74 *^,■■^	33.3 ± 3.11 *^,■■■^	36.4 ± 3.20 *	38.5 ± 3.71 *	38.4 ± 3.68 *
P30	9.90 ± 1.21	19.2 ± 1.73 *	32.3 ± 2.90 *^,●^	40.2 ± 3.10 *^,●●^	50.0 ± 4.69 *^,●●●^	53.7 ± 4.78 *^,▲^	55.6 ± 5.22 *^,▲^	55.3 ± 5.09 *^,▲^

TR2 = Mitochondrial Thioredoxin Reductase. Values shown are means ± SD of duplicate determinations for 6 cultures. MEM1 = routine growth medium. MEM2–MEM8 = FBS-free media supplemented with Se as detailed in text. * = *p* < 0.0001 when comparing TR2 activities in P20 and P30 subcultures incubated with MEM2–MEM8 against those recorded for the same cells incubated with MEM1. ^■^ = *p* < 0.0001, ^■■^ = *p* <0.01, ^■■■^ = *p* < 0.05 when comparing TR2 activities in P20 subcultures incubated in MEM3 against MEM2, MEM4 against MEM3, and MEM5 against MEM4, respectively. Such comparisons performed between MEM6, 7, and 8 were not significant. ^●^ = *p* < 0.0001, ^●●^ = *p* < 0.001, ^●●●^ = *p* < 0.01 when comparing TR2 activity in P30 subcultures incubated in MEM3 against MEM2, MEM4 against MEM3, and MEM5 Against MEM4, respectively. Such comparisons performed between cells incubated in MEM6, 7, and 8 were not significant. ^▲^ = *p* < 0.0001 when comparing TR2 activities in P30 cells incubated with MEM6, 7 and 8 against those recorded for P20 cells incubated with the same media.

**Table 4:** Effect of Se supplementation on mitochondrial H_2_O_2_ generation rates in pre-senescent (P5) and senescent (P20 and P30) fibroblasts subcultures.

Passage of Subculture (*n* = 6)	H_2_O_2_ Generation (pmol/min/mg Protein)							
	MEM1	MEM2	MEM3	MEM4	MEM5	MEM6	MEM7	MEM8
P5	3.41 ± 0.29	3.20 ± 0.28	3.52 ± 0.30	3.58 ± 0.31	3.35 ± 0.30	3.40 ± 0.28	3.61 ± 0.32	3.33 ± 0.28
P20	7.80 ± 0.63	7.12 ± 0.58	6.54 ± 0.53 *	6.03 ± 0.50 **	5.62 ± 0.47 **	5.15 ± 0.42 **	4.64 ± 0.38 **	4.28 ± 0.34 **
P30	13.8 ± 1.14	10.3 ± 0.83 **	9.10 ± 0.74 **^,■^	8.10 ± 0.65 **^,■^	6.99 ± 0.58 **^,■^	5.69 ± 0.49 **^,■■^	4.78 ± 0.40 **^,■■^	4.22 ± 0.35 **^,■^

H_2_O_2_ = Hydrogen peroxide. Values are means ± SD of duplicate determinations for 6 cultures. MEM1 = routine growth medium. MEM2-MEM8 = FBS-free media supplemented with Se as detailed in text. * = *p* < 0.01, ** = *p* < 0.0001 when comparing H_2_O_2_ generation rates in P20 and P30 subcultures incubated with MEM2–MEM8 against that recorded for the same cells incubated with MEM1. ^■^ = *p* < 0.05, ^■■^ = *p* < 0.01 when comparing H_2_O_2_ generation rates in P30 subcultures incubated in MEM3 against MEM2, MEM4 against MEM3, MEM5 against MEM4, MEM6 against MEM5, MEM7 against MEM6, and MEM8 against MEM7. Such comparisons performed for P20 subcultures were not significant.
